# Use of Tobacco Tax Stamps to Prevent and Reduce Illicit Tobacco Trade — United States, 2014

**Published:** 2015-05-29

**Authors:** Jamie Chriqui, Hillary DeLong, Camille Gourdet, Frank Chaloupka, Sarah Matthes Edwards, Xin Xu, Gabbi Promoff

**Affiliations:** 1 Division of Health Policy and Administration and Institute for Health Research and Policy, School of Public Health, University of Illinois at Chicago; 2 Institute for Health Research and Policy, School of Public Health, University of Illinois at Chicago; 3 Department of Economics and Institute for Health Research and Policy, School of Public Health, University of Illinois at Chicago; 4 Office on Smoking and Health, National Center for Chronic Disease Prevention and Health Promotion, CDC

Tobacco use is the leading cause of preventable disease and death in the United States (1). Increasing the unit price on tobacco products is the most effective tobacco prevention and control measure (2). Illicit tobacco trade (illicit trade) undermines high tobacco prices by providing tobacco users with cheaper-priced alternatives (3). In the United States, illicit trade primarily occurs when cigarettes are bought from states, jurisdictions, and federal reservation land with lower or no excise taxes, and sold in jurisdictions with higher taxes. Applying tax stamps to tobacco products, which provides documentation that taxes have been paid, is an important tool to combat illicit trade. Comprehensive tax stamping policy, which includes using digital, encrypted (“high-tech”) stamps, applying stamps to all tobacco products, and working with tribes on stamping agreements, can further prevent and reduce illicit trade (4,5). This report describes state laws governing tax stamps on cigarettes, little cigars (cigarette-sized cigars), roll-your-own tobacco (RYOT), and tribal tobacco sales across the United States as of January 1, 2014, and assesses the extent of comprehensive tobacco tax stamping in the United States. Forty-four states (including the District of Columbia [DC]) applied traditional paper (“low-tech”) tax stamps to cigarettes, whereas four authorized more effective high-tech stamps. Six states explicitly required stamps on other tobacco products (i.e., tobacco products other than cigarettes), and in approximately one third of states with tribal lands, tribes required tax stamping to address illicit purchases by nonmembers. No U.S. state had a comprehensive approach to tobacco tax stamping. Enhancing tobacco tax stamping across the country might further prevent and reduce illicit trade in the United States.

The Tobacconomics Program* examined state statutes and regulations and, for tribal tobacco sales, relevant agency opinions and case law, under a cooperative agreement funded by the National Cancer Institute as part of its State and Community Tobacco Control Initiative, 2011–2015. State laws were compiled through primary legal research using the Westlaw and Lexis-Nexis commercial legal research services. Where possible, state law data were verified against publicly available secondary sources, including CDC’s State Tobacco Activities Tracking and Evaluation system,† which provides current and historical state-level data on tobacco use prevention and control, including cigarette stamping. Clarification of codified law was sought through state or federal case law, Attorneys General opinions, and notices or rulings from states’ departments of revenue. Excluded from the tribal sales research were state laws that made general reference to tobacco sales without explicit reference to tribes or application to tribal sales by case law, Attorneys General opinions, or departments of revenue notices; also excluded were tribal codes, tax agreements, or compacts not codified by the state (i.e., individual tribe-specific codes and policies).

As of January 1, 2014, a total of 48 states (including DC) applied cigarette tax stamps. Only four of these authorized the use of high-tech stamps. Three of these four states (California, Massachusetts, and Michigan) have implemented their use; New Jersey has not (Table). Of the 17 states that taxed little cigars at an amount equivalent to cigarettes, which makes them subject to stamping, only five of these states’ laws explicitly required stamps on little cigars. Of the five states that taxed RYOT as cigarettes, which makes them subject to stamping, only two explicitly required stamps on RYOT (Table, Figure 1).

Although Native American tribes within the United States are protected by sovereign immunity and states do not have legal authority over tribes within their borders, agreements, such as ones to regulate tobacco sales, may be negotiated. Thirty-four states have federal reservation land within their borders. Of these, 20 regulated tribal tobacco sales as of January 1, 2014, 13 of which explicitly addressed stamping of products sold on-reservation (Table, Figure 2). Of those 13, nine required stamps on all cigarettes or tobacco products sold on-reservation, and four only required stamps on products sold to nonmembers of the tribe or on all products sold by tribes without tax agreements with the state.

## Discussion

This report indicates that although the majority of states required low-tech cigarette tax stamps as of January 1, 2014, few were using high-tech stamps, applying stamps to other tobacco products, or working with tribes on stamping agreements. Depending on analytical approaches and definitions of illicit trade, it is estimated that 8%–21% of cigarettes consumed in the United States are purchased illicitly (4). These illicit purchases undermine tobacco control efforts (2), might contribute to health disparities (4), and reduce local and state revenues by billions of dollars annually (4). Lack of comprehensive tax stamping could thwart U.S. efforts to reduce illicit trade and complicate law enforcement.

Three states (North Carolina, North Dakota, and South Carolina) did not require any stamps, making tax collection more difficult and potentially facilitating illicit trade. The majority of states use low-tech stamps on cigarettes, which are easier to counterfeit (6). These conventional stamps do not take advantage of overt and covert security features and encrypted information regarding manufacturing, distribution, and retail destination (4) that is contained in high-tech stamps. A recent study of littered cigarette packs in New York City found that approximately 60% of packs examined lacked the appropriate tax stamp (7), which was more prevalent in socioeconomically deprived areas, suggesting that illicit trade might exacerbate existing health disparities by facilitating access to cigarettes and making them more affordable to persons with lower incomes (7).

A few states are successfully employing high-tech stamps (4). Anti-counterfeit technology enables enforcement agents to immediately authenticate the stamp and to detect counterfeit stamps. A study in California showed that the additional tax revenues collected using the state’s high-tech stamp could be as much as eight times higher than implementation and administrative costs (4).

Although most states applied at least low-tech stamps to cigarettes, only a few expressly stamped little cigars or RYOT. Requiring stamps on other tobacco products, especially cigarette analogues such as little cigars and RYOT, is an important aspect of preventing tax avoidance by minimizing opportunities and incentives for substitution (2). Without stamps, it is difficult for inspectors to distinguish tobacco products on which tax has been paid from those coming from illicit markets.

A critical facet of a comprehensive approach to tobacco stamping is the inclusion of all sources of tobacco in this practice, including sales by Native American tribes. Several states have entered into agreements with Native American tribes on general tobacco-related issues or have negotiated specific tax agreements with tribes to reduce the avoidance of tobacco excise taxes by nonmembers, including application of tax stamps to products sold on-reservation. Although tribal members who purchase tobacco on-reservation are exempt from state taxation, nonmembers purchasing on-reservation are not exempt from state taxation; these illegal purchases by nonmembers are a significant source of illicit trade because of challenges in collecting taxes on sales to nonmembers (8). Agreements requiring stamp application or a state’s decision to apply stamps strategically within the distribution chain might alleviate concerns about tax losses from tribal sales, because it encourages prepayment of taxes, and might aid in enforcement of excise tax payment by establishing clear procedures and tax rates for products sold on federal reservation land.

What is already known on this topic?

Increasing the unit price on tobacco products is the most effective tobacco prevention and control intervention, especially among price-sensitive populations, such as youth. Illicit tobacco trade can undermine the effectiveness of high tobacco prices by providing tobacco users with cheaper priced alternatives. Tobacco tax stamping is intended to further support efforts to prevent and reduce illicit trade.

What is added by this report?

A comprehensive tax stamping approach includes the use of digital, encrypted (“high-tech”) stamps, the application of stamps to all tobacco products, including little cigars and roll-your-own tobacco; and working with Native American tribes on stamping agreements. As of January 1, 2014, most states used traditional paper (“low-tech”) stamps that are easy to counterfeit, and many did not explicitly require stamps on cigarette-equivalent products such as little cigars and roll-your-own tobacco. Approximately two thirds of states with federal reservation land did not have codified agreements that permit tobacco stamping of tribally sold products.

What are the implications for public health practice?

Illicit trade undermines tobacco control efforts and might contribute to health disparities. Comprehensive tax stamping policies could enhance U.S. efforts to reduce illicit trade, thereby increasing revenues as well as protecting public health and reducing smoking by stopping illegal cigarette sales.

The findings in this report are subject to at least three limitations. First, the cigarette, little cigar, and RYOT data were limited to codified statutory and administrative law and do not include Attorneys General opinions, case law, or departments of revenue–issued notices, rulings, or decisions. For example, California’s statutes or regulations do not explicitly call for little cigar stamping. However, per a notice issued by California’s Board of Equalization (excluded from this report’s primary legal research), all little cigars must be stamped.§ Second, this report did not include information on states that maintain general tobacco sales laws that are not explicitly enforced with tribal entities, and it was not possible to determine whether the states that regulate tribal tobacco sales, but do not explicitly address stamping do, in fact, include stamps in their noncodified agreements or compacts. In addition, a tribe’s own laws might dictate tribal tax rates or enforcement mechanisms not captured in this report. Finally, this report only reviewed the laws pertaining to the use of tax stamps on tobacco products; however, tax stamping on its own is not sufficient to deter illicit trade. Enforcement is also necessary (5,6). Other policy interventions, such as licensing, implementing a track-and-trace system, and the harmonization of tax codes, also contribute to reductions in illicit trade (3).

A comprehensive approach to tobacco tax stamping could be an important tool in reducing illicit trade and revenue loss in the United States. Applying tax stamps to all tobacco products, and for those states with federal reservation land within their borders, working with tribes to negotiate mutually beneficial agreements, including the use of stamps on tobacco products sold on reservation land, could have an important impact on reducing illicit trade and further reduce smoking and associated health care costs as well as recoup lost revenues from illicit trade (4). Additionally, introducing high-tech tax stamps with new technologies including encryption, holograms, and scannable barcodes in all states could further reduce counterfeiting and improve supply-chain monitoring and enforcement (4).

## Figures and Tables

**FIGURE 1:**
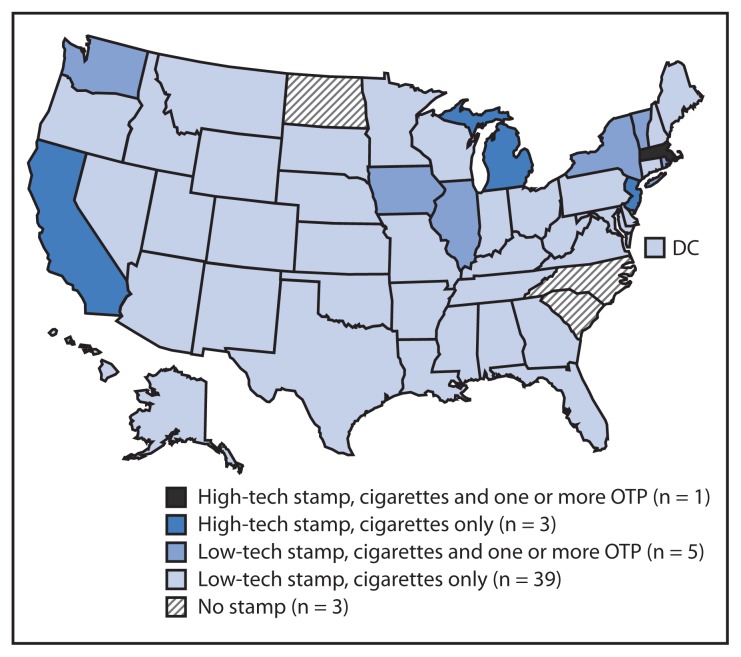
Use and type of cigarette and other tobacco product (OTP) stamps, by state — United States, January 1, 2014 **Source:** Tobacconomics Program, Health Policy Center, Institute for Health Research and Policy, University of Illinois at Chicago. Additional information available at http://www.tobacconomics.org.

**FIGURE 2:**
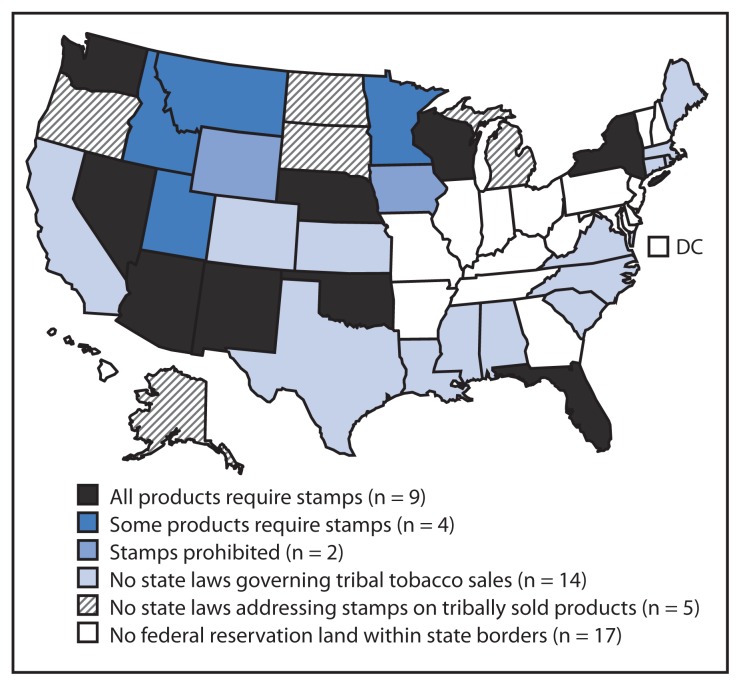
Laws governing use of tobacco stamps on tobacco products sold on tribal reservations, by state — United States, January 1, 2014 **Source:** Tobacconomics Program, Health Policy Center, Institute for Health Research and Policy, University of Illinois at Chicago. Additional information available at http://www.tobacconomics.org.

**TABLE:** States with laws requiring tax stamps on cigarettes, little cigars (LC), roll-your-own tobacco (RYOT), and tribal tobacco — United States, January 1, 2014

	Cigarettes		LC and RYOT		Tribal stamping	
			
State (and District of Columbia)	Stamp required	Encrypted tax stamp	LC and/or RYOT taxed as a cigarette*	LC and/or RYOT explicitly stamped	On-reservation tobacco sales require stamps on some or all products	Type of stamp(s) required
Alabama	Yes					
Alaska	Yes				—†	
Arizona	Yes				Yes (all§)	SE, GT, O¶
Arkansas	Yes		RYOT			
California	Yes	Yes	LC			
Colorado	Yes					
Connecticut	Yes					
Delaware	Yes					
District of Columbia	Yes		LC			
Florida	Yes				Yes (all§)	Silent**
Georgia	Yes					
Hawaii	Yes		LC			
Idaho	Yes				Yes (some††)	SE
Illinois	Yes		LC	LC		
Indiana	Yes					
Iowa	Yes		LC	LC	Prohibited§§	
Kansas	Yes					
Kentucky	Yes					
Louisiana	Yes					
Maine	Yes					
Maryland	Yes					
Massachusetts	Yes	Yes	LC	LC		
Michigan	Yes	Yes			—†	
Minnesota	Yes		LC		Yes (some††)¶¶***	SE, TA
Mississippi	Yes					
Missouri	Yes					
Montana	Yes		LC		Yes (some††)***	SE
Nebraska	Yes				Yes (all§)	SE, ST
Nevada	Yes				Yes (all§)	SE, GT
New Hampshire	Yes		LC, RYOT			
New Jersey	Yes	Yes†††				
New Mexico	Yes		LC, RYOT		Yes (all§)	SE, GT§§§
New York	Yes		LC		Yes (all§)	SE
North Carolina	No					
North Dakota	No				—†	
Ohio	Yes					
Oklahoma	Yes				Yes (all§)	SE, GT, TA
Oregon	Yes				—†	
Pennsylvania	Yes		LC			
Rhode Island	Yes		LC	LC		
South Carolina	No		LC			
South Dakota	Yes				—†	
Tennessee	Yes					
Texas	Yes					
Utah	Yes		LC		Yes (some††)¶¶	SE
Vermont	Yes		LC, RYOT	LC, RYOT		
Virginia	Yes					
Washington	Yes		LC, RYOT	RYOT	Yes (all§)	SE, ST, TA
West Virginia	Yes					
Wisconsin	Yes				Yes (all§)	SE, GT
Wyoming	Yes				Prohibited§§	
**Totals**	**48**	**4**	**18**	**6**	**13**	**—**

**Source:** Tobacconomics Program, Health Policy Center, Institute for Health Research and Policy, University of Illinois at Chicago. Additional information available at http://www.tobacconomics.org.

**Abbreviations:** SE = state excise stamp; GT = general tribal stamp (used by all tribes); O = other; TA = tribal agreement stamp (used by all tribes with tribal agreement); ST = specific tribal stamp (specific to certain tribe).

* In these states, LC and/or RYOT are taxed as cigarettes and, therefore, with the exception of LC in South Carolina (where cigarettes are not stamped), might be subject to cigarette stamping requirements.

† State regulates tribal tobacco sales but is silent on the stamping issue.

§ State laws explicitly state that all cigarettes or tobacco products sold on-reservation require stamps.

¶ Tax-free reservation stamp.

** Law is silent on specific stamps required for tribal sales.

†† In certain instances (e.g., products sold to nonmembers or products sold to tribes without tax agreements), cigarettes or tobacco products sold on-reservation require stamps.

§§ Stamps explicitly prohibited on cigarettes or tobacco products sold on-reservation.

¶¶ Tax stamps required on products sold to nonmembers.

*** Tax stamps required on products sold to tribes without agreements.

††† Authorized by law but not currently implemented.

§§§ New Mexico has a general tribal tax-exempt stamp (for tribal members) and a tax credit stamp (for sales to nonmembers on reservation).

## Footnotes

* Tobacconomics Program, Health Policy Center, Institute for Health Research and Policy, University of Illinois at Chicago. Additional information available at http://www.tobacconomics.org.

† Information available at http://www.cdc.gov/tobacco/data_statistics/state_data/state_system/index.htm.

§ Information available at http://www.boe.ca.gov.
